# Monoamine Oxidase Inhibition by Plant-Derived β-Carbolines; Implications for the Psychopharmacology of Tobacco and Ayahuasca

**DOI:** 10.3389/fphar.2022.886408

**Published:** 2022-05-02

**Authors:** Ilana Berlowitz, Klemens Egger, Paul Cumming

**Affiliations:** ^1^ Department of Nuclear Medicine, Inselspital Bern University Hospital, University of Bern, Bern, Switzerland; ^2^ Department of Psychiatry, Psychotherapy and Psychosomatics, Psychiatric University Hospital Zurich, University of Zurich, Zurich, Switzerland; ^3^ School of Psychology and Counselling, Queensland University of Technology, Brisbane, QLD, Australia

**Keywords:** tobacco, *Nicotiana*, ayahuasca, *Banisteriopsis caapi*, monoamine oxidase (MAO), β-carbolines, harmine, dimethyltryptamine (DMT)

## Abstract

The monoamine oxidases (MAOs) are flavin-containing amine oxidoreductases responsible for metabolism of many biogenic amine molecules in the brain and peripheral tissues. Whereas serotonin is the preferred substrate of MAO-A, phenylethylamine is metabolized by MAO-B, and dopamine and tyramine are nearly ambivalent with respect to the two isozymes. β-Carboline alkaloids such as harmine, harman(e), and norharman(e) are MAO inhibitors present in many plant materials, including foodstuffs, medicinal plants, and intoxicants, notably in tobacco (*Nicotiana* spp.) and in *Banisteriopsis caapi*, a vine used in the Amazonian ayahuasca brew. The β-carbolines present in *B. caapi* may have effects on neurogenesis and intrinsic antidepressant properties, in addition to potentiating the bioavailability of the hallucinogen *N,N*-dimethyltryptamine (DMT), which is often present in admixture plants of ayahuasca such as *Psychotria viridis*. Tobacco also contains physiologically relevant concentrations of β-carbolines, which potentially contribute to its psychopharmacology. However, in both cases, the threshold of MAO inhibition sufficient to interact with biogenic amine neurotransmission remains to be established. An important class of antidepressant medications provoke a complete and irreversible inhibition of MAO-A/B, and such complete inhibition is almost unattainable with reversible and competitive inhibitors such as β-carbolines. However, the preclinical and clinical observations with synthetic MAO inhibitors present a background for obtaining a better understanding of the polypharmacologies of tobacco and ayahuasca. Furthermore, MAO inhibitors of diverse structures are present in a wide variety of medicinal plants, but their pharmacological relevance in many instances remains to be established.

## Introduction

**FIGURE 1:**
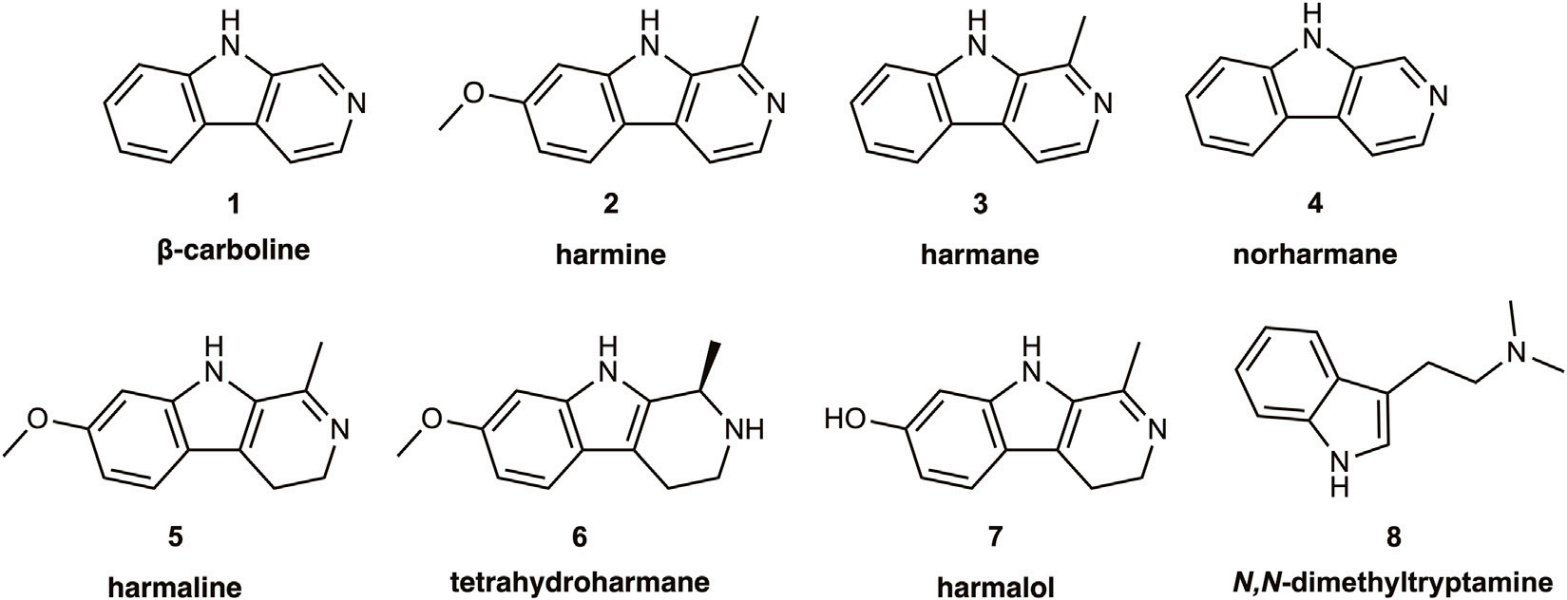
Structures of the alkaloid β-carboline, its common naturally occurring derivatives and metabolites, as well as of the hallucinogen *N,N*-dimethyltryptamine.

The monoamine oxidases (MAOs EC 1.4.3.4) are flavin-containing amine oxidoreductases that occur in the outer mitochondrial membrane of most mammalian cells. MAOs deaminate their endogenous substrates in the presence of molecular oxygen; this generates the corresponding aldehyde-intermediates, which further metabolize to carboxylic acids or alcohol derivatives. The deamination step releases the amine nitrogen as ammonia and with the production of hydrogen peroxide. As such, MAO is a double-edged sword, ridding the organism of excess biogenic monoamine compounds, while also producing potentially toxic metabolites. MAO occurs in MAO-A and MAO-B isozymes with slightly divergent amino acid sequences, and having similar genetic organization with 15 exons on the X-chromosome (Shih and Chen, 2004). Apparently having arisen by a gene duplication event early in the mammalian lineage, the two forms have differing substrate specificities. Whereas serotonin is the preferred substrate of MAO-A, phenylethylamine is the preferred substrate for MAO-B; dopamine and tyramine are nearly ambivalent with respect to the two forms of MAO (Schoepp and Azzaro, 1981; Yang and Neff, 1973). These metabolic activities point to a key role of MAO in maintaining homeostasis of biogenic monoamine neurotransmitters, but by no means does MAO expression occur only in the central nervous system (CNS); MAO-A is present in the liver, pulmonary vasculature, the gastrointestinal tract (more about this later) and the placenta, whereas MAO-B is present in blood platelets. As shall be seen below, heterocyclic β-carboline (9H-pyrido[3,4-b]indole) alkaloids such as harmine, harmane, and norharmane^1^ are MAO inhibitors present in many plant materials, including tobacco and its smoke, and in the decoctions known as ayahuasca or yagé used in Indigenous Amazonian medical systems and practices. In the context of the psychopharmacology of ayahuasca, it is widely believed that β-carbolines may potentiate the psychoactive potency of *N,N*-dimethyltryptamine (DMT); although from a traditional Amazonian perspective this may not be the only essential mechanism of action, and ayahuasca brew samples need not invariably contain DMT (McKenna 1984a; Callaway et al., 2005; Ona et al., 2020; Politi et al., 2021), we present for reference purposes the chemical structures of DMT and key naturally occurring β-carbolines (Figure 1).

## The Classes of MAO Inhibitors

MAO inhibitors fall into various categories with respect to the nature of the binding to the enzyme. Certain molecules are MAO substrates that can exert competitive inhibition of the enzyme by virtue of their occupation of the binding pocket, and other compounds, like the β-carbolines, are reversibly binding competitive inhibitors that are not substrates for MAO. Still other compounds, like pargyline, clorgyline, and rasagiline, are irreversible MAO inhibitors, also known as suicide substrates. This class of MAO inhibitor are substrates that mechanistically form a covalent bond with the enzyme via the propargyl moiety; this reaction permanently kills the enzyme. To this day, there is a prevailing notion that pharmacologically blocking the breakdown of dopamine, serotonin, and other MAO substrates should underlie the antidepressant property of MAO inhibitors by rectifying some neurochemical deficiency, despite the paucity of evidence for any such deficiency syndrome in patients with depression (Gryglewski, 2014). Be that as it may, irreversible MAO inhibitors were among the first effective antidepressant medications (Quitkin et al., 1979), including the non-selective (MAO-A/B) inhibitors phenelzine (Nardil), isocarboxazid (Thase et al., 1995), and tranylcypromine (Ulrich et al., 2017). While effective against depression and anxiety symptoms, the irreversible MAO inhibitors have fallen out of favor due to the risk of toxic interactions arising from complete blockade of MAO. Certain foods and beverages contain high levels of pharmacologically active substances such as tyramine that normally undergoes inactivation by MAO-A in the gut. Consuming certain cheeses rich in tyramine can thus provoke a dangerous hypertensive crisis in people treated with irreversible MAO inhibitors, known as the “cheese effect” (Youdim and Weinstock, 2004). While individuals free of MAO inhibition can consume tyramine by the spoonful without ill effect, patients treated with tranylcypromine are at risk for a hypertensive crisis after consuming as little as 10 mg of tyramine (Gillman, 2011; Ulrich et al., 2017). This is unlikely to occur with use of irreversible MAO-B inhibitors such as rasagiline (an antiparkinsonian compound), or with reversible MAO-A inhibitors such as moclobemide, an antidepressant that has largely supplanted irreversible inhibitors in clinical practice, despite arguments for their greater antidepressant efficacy (Shulman et al., 2013). In addition to the perhaps overstated cardiovascular risks, MAO inhibition has effects on the chemistry of the CNS. For example, treatment with pargyline increased the vesicular concentrations of dopamine and noradrenaline in rodent brain (Buu and Lussier, 1989). Acute treatment with pargyline enormously increases the brain concentrations of the trace amines phenylethylamine and tryptamine (Durden and Philips, 1980) as well as tyramine (Suzuki et al., 1979) and other MAO substrates that are normally present at low concentrations in brain. There is a diverse family of trace amine-associated receptors (TAAR), which can exert a variety of actions in the brain and peripheral tissues (Gainetdinov et al., 2018). It follows that treatment of patients with MAO inhibitors should exert actions extending beyond effects on the far more abundant neurotransmitters such as dopamine, noradrenaline, and serotonin.

## The Behavioral Effects and Neurochemistry of MAO Inhibition

While MAO inhibition should increase the levels of a wide variety of endogenous and xenobiotic substances, the threshold for physiologically relevant MAO inhibition is not well established. In particular, is MAO inhibition from tobacco smoke merely an epiphenomenon, or does it contribute to the psychopharmacology of tobacco use, its experienced effects, and/or dependence? Some work indicates that the norharmane level in plasma of habitual smokers was inversely related to tobacco craving in smokers with a relatively low dependence on tobacco (Van Den Eijnden et al., 2003). This suggests that certain β-carbolines may have intrinsic effects on drug-seeking behavior, unrelated to any direct interaction with (for example) nicotine. Mice treated with the irreversible MAO inhibitor tranylcypromine showed an increased liking for nicotine, based on their self-administration behavior (Villégier, 2007). Similar studies in rats showed that clorgyline or norharmane (5 mg⁄kg⁄day) potentiated the reinforcing effects of nicotine, whereas the MAO-B selective inhibitor *L*-deprenyl had no such effect (Guillem, 2006). Yet another study confirmed the potentiation of nicotine self-administration in rats pretreated with tranylcypromine, but failed to show a comparable effect from a cocktail of tobacco alkaloids including harman and norharmane, perhaps due to insufficient dose (Smith, 2015). On the other hand, treatment of rats with three β-carbolines (harmane, norharmane, and harmine) evoked dose-dependent increases in the threshold for intracranial self-stimulation (ICSS) (Harris et al., 2020). In this experimental paradigm, trained rats press a lever in order to get a (rewarding) electrical stimulation of mesolimbic dopamine release. The increasing ICSS thresholds suggest that β-carbolines in isolation exerted aversive/anhedonic effects. Conditioned place preference (CPP) is another paradigm to measure “drug-liking”; if an animal finds a drug treatment rewarding, they will tend to return to the very spot where the drug was first administered, which might be described as magical thinking, or simple pragmatism. Pretreatment of rats with clorgyline at a low dose (1 mg/kg) did have some interaction with the CPP induced by the powerful psychostimulant methamphetamine (Kitanaka, 2006), and a still lower clorgyline dose (0.1 mg/kg) more clearly interfered with the methamphetamine CPP, while selectively decreasing the concentration in brain of the noradrenaline metabolite 3-methoxy-4-hydroxyphenylglycol (MHPG) (Kitanaka, 2010). Thus, partial MAO inhibition in brain may be relevant to the reinforcing and/or aversive properties of drugs such as nicotine or methamphetamine.

Genetic ablation of MAO gives insight into the perturbations produced by pharmacological MAO inhibition. Thus, knockout of MAO-A in mice results in elevated brain levels of dopamine, serotonin, and noradrenaline (Shih and Chen, 1999), along with a behavioral phenotype including aggression, perseveration, and social behavior deficits, which have been likened to autism spectrum disorder (Bortolato, 2013), and which were partially rescued by treatment with serotonin reuptake inhibitors (Godar, 2014). Perhaps similarly, males in a Dutch kindred with a loss of function mutation in the MAO-A gene showed a pattern of intellectual disability as well as abnormal behavior including aggression, arson, and various other forms of criminality (Brunner, 1993). However, mice with ablation of the MAO-B gene do not show the aggressive behavior typical of MAO-A knockouts, but have the expected increases in tissue levels of phenylethylamine (Shih and Chen, 1999). Two individuals with Norrie disease involving deletion of the MAO sequences on the X-chromosome showed markedly reduced formation of the noradrenaline metabolite MHPG and enormously elevated urine levels of the MAO-B substrates phenylethylamine and tyramine (Murphy, 1990). In some sense, these developmental abnormalities may mirror the consequences of pharmacological MAO inhibition, with the caveat that genetic ablation must result in a divergent pathway of brain development.

How might MAO inhibition alter cerebral function by increasing levels of neurotransmitters? The cerebral microdialysis technique enables the detection of dynamic changes in interstitial neurotransmitter levels after drug treatments and across behavioral states. In one such study, treatment with a high dose of pargyline non-significantly increased the interstitial level of dopamine in rat striatum by about 50% (Cumming, 1992), while enormously increasing the levels of the default metabolite 3-methoxytyramine (Brown, 1991). A subsequent study seemed to show that pargyline-evoked increases in dialysates from rat striatum were likely an artefact of the procedure for implanting the dialysis probes (Blaha et al., 1996). We may suppose that dopamine fibers autoregulate their rate of dopamine synthesis and release following MAO inhibition, despite the net increased tissue concentration of dopamine. While dopamine is ambivalent with respect to the form of MAO *in vitro*, other microdialysis studies involving clorgyline or *L*-deprenyl treatment indicate that MAO-A normally metabolizes dopamine in mouse striatum, but that MAO-B also contributes under conditions of elevated dopamine levels (Fornai, 1999). This may not hold for the rat, in which brain dopamine metabolism is apparently exclusively mediated by MAO-A; the case is less clear for humans, in whom the expression of MAO-B increases with age (Shih et al., 1999). Various microdialysis studies showed that interstitial serotonin levels were two-fold higher in MAO-A knockout mice compared to wild-type mice, and that other perturbations included down-regulation of serotonin transporters and loss of autoregulation of serotonin release. These adaptive phenomena might underlie the antidepressant effects of irreversible MAO blockers, but the incomplete inhibition obtained with plant-derived MAO inhibitors may not suffice to produce chronic adaptive changes in dopamine and serotonin pathways. On the other hand, the presence of 2,3,6-trimethyl-1,4-naphthoquinone and β-carbolines in tobacco smoke may be responsible for the protection against Parkinson’s disease afforded by smoking. A follow-up study of 30,000 male British doctors lasting 65 years indicated a 30–50% lower incidence of Parkinson’s disease among the smokers (Mappin-Kasirer, 2020). As in the case of 1-methyl-4-phenyl-1,2,3,6-tetrahydropyridine (MPTP)-induced neurotoxic parkinsonism, uninhibited MAO may generate a toxic metabolite or excess amounts of hydrogen peroxide, thus promoting the senescence of vulnerable dopamine neurons (Castagnoli and Murugesan, 2004).

## Natural Sources of MAO Inhibitors; the Case of Tobacco

Substances acting as MAO inhibitors occur in a wide variety of lichen, fungi, and plants. For example, 5-hydroxy-2-methyl-chroman-4-one from a lichen fungus (*Daldinia fissa*) inhibits MAO-A/B at µM concentrations (Jeong, 2021), as do a variety of plant-derived and synthetic coumarin derivatives (Koyiparambath, 2021). The pigment derived from madder root (purpurin; 1,2,4-trihydroxyanthraquinone) is another MAO inhibitor with reputed antidepressant effects in a rodent model (Ma, 2020). Even licorice (*Glycyrrhiza glabra*) extracts are significantly potent inhibitors of MAO-B *in vitro* (Ramadan, 2021), perhaps due to the sweet tasting saponin compound glycyrrhizin. St. John’s Wort (*Hypericum perforatum*) has long served as an antidepressant in traditional medicine, an effect often attributed to MAO-inhibition by one or more of its constituents. Floral extracts indeed inhibited MAO-A *in vitro* (IC_50_ 65 μg/ml), which proved to be due to quercetin and flavonoids rather than hypericin (Herraiz and Guillén, 2018), much as shown in an earlier study (Bladt and Wagner, 1994); the more recent study indicated 1000-fold higher inhibition of MAO-A by *Peganum harmala* extracts, which contain high levels of harmaline and harmine. MAO-inhibitory activity in a plant extract need not suffice to evoke significant MAO inhibition upon consumption. However, concentrations of the β-carboline alkaloids harmane and norharmane in sesame seeds or their oil may partially inhibit MAO after consumption (Liu, 2021).

Recent years has seen burgeoning interest in the pharmacological and therapeutic effects of β-carbolines, which are present at significant concentrations in extracts of *P. harmala*, a rue native to the Mediterranean region (Moloudizargari, 2013), and in the South American vine *Banisteriopsis caapi*. The latter plant is the key constituent of the ayahuasca decoction widely used in Indigenous Amazonian social and medical practice. In that context, MAO-inhibition among other things serves to potentiate central action of the hallucinogenic component *N,N*-dimethyltryptamine (DMT), which is a hallucinogenic serotonin 5-HT_2A_ agonist otherwise subject to rapid deamination in the gut following oral administration (Cumming, 2021), as attested by a series of self-experiments (Ott, 1999). DMT also forms endogenously in the living brain and might thus properly be termed a trace amine neurotransmitter comparable to tyramine, phenylethylamine, and tryptamine. The latter trace amines normally occur at very low concentrations in brain, being vulnerable to rapid metabolism by MAO; it is currently unknown if MAO inhibition increases the brain concentration of endogenous DMT to a relevant extent. Certainly, irreversible MAO inhibitors are not hallucinogenic, but their administration might still increase brain levels of DMT to a degree evoking some physiological effects or interactions. However, DMT is naturally present in a wide variety of plant sources; notably the south American plant *Psychotria viridis* (Carbonaro and Gatch, 2016), and likewise reportedly in *Diploterys cabrerana* (McKenna 1984) and *Mimosa tenuiflora* (Simão, 2019). While orally administered DMT is essentially inactive, conjoint consumption of the ayahuasca decoction’s constituents (e.g., *B. caapi* and *P. viridis*) (Simão, 2019), evokes an intense psychedelic and visionary experience. However, these two plants have highly variable alkaloid contents; 32 *B. caapi* samples collected in Brazil had β-carboline concentrations ranging from 0.3 to 8.4 mg/g dry weight for harmine and 0.03–8.3 mg/g dry weight for harmaline (Callaway et al., 2005). In the same study 36 samples of *P. viridis* had DMT contents ranging from nil to 18 mg/g dry weight. Other constituents of ayahuasca rituals may sometimes include the potent tobacco species *Nicotiana rustica*, which contains unknown levels of β-carbolines and other alkaloids. Hallucinogenic preparations of myristicaceous bark and leaf samples contained DMT and its congener 5-methoxy-N,N-dimethyltryptamine (5-MeO-DMT), but only traces of β-carbolines (McKenna, 1984b), an observation which may call for qualifying the necessity of conjoint administration of DMT along with an MAO inhibitor.

According to Paracelsus, *dosis sola facit venenum* (the dose makes the poison). In our considerations of MAO interactions, we must be mindful that MAO inhibitors, despite their ubiquity in plants, may not be sufficiently active to have pronounced effects on biogenic amine metabolism in the living organism. For example, sensitive analyses reveal the presence of harmane and norharmane at low concentrations (<50 ng/g) in a wide variety of foodstuffs, and moderately high levels in coffee beverages (200 ng/ml). The first attestation of the presence of harmane and norharmane in tobacco, and its apparent pyrolytic formation from tryptophan in the tobacco, dates to 1962 (Poindexter and Carpenter, 1962). Janiger and de Rios likewise noted 40–100-fold higher harmane and norharmane levels in tobacco smoke than in the uncombusted tobacco and raised the possibility of inherent psychoactive effects of these compounds (Janiger and de Rios, 1973). More recent reports indicate impressively high concentrations of these alkaloids in cigarette smoke (up to 3,000 ng/cigarette) (Herraiz, 2004). In keeping with the notion of pyrogenic formation of β-carbolines, others reported concentrations of around 0.5 ng/g harmaline and harmine in the leaves and flowers of *Nicotiana tabacum* (Tarkowská, 2020). Interestingly, the authors of that study also reported relatively high concentrations (10 ng/g) of the alpha_2_ antagonist yohimbine, which has a pressor and anxiogenic action; administration of yohimbine increased the reinforcing efficacy nicotine in an intravenous self-administration paradigm, but only in female rats (Li, 2014).

As noted above, mainstream smoke from commercial tobacco contains roughly 2 µg harmane and 4 µg norharmane, but smoke from barley tobacco can have several-fold higher concentrations (Zhang et al., 2011). The authors of that study also reported on the presence in smoke of “exotic” heterocyclic compounds derived from tryptophan, namely 3-amino-1,4-dimethyl-5H-pyrido[4,3-b]indole and 3-amino-1-methyl-5H-pyrido[4,3-b]indole. Tobacco smoke particulate has very high nicotine concentrations, greatly exceeding the amounts of harmane and norharmane, which seemed insufficient to inhibit completely MAO-A/B in smokers (Truman et al., 2017); the authors postulated that unidentified substances might account better for the MAO-inhibitory effect of smoking. Nonetheless, β-carbolines such as norharmane are clearly present in blood of tobacco smokers, attaining a concentration of 200 pg/ml after smoking a single cigarette (Breyer-Pfaff, 1996). Further work demonstrated an association with smoking, but no such effect of alcohol consumption by non-smokers (Spijkerman, 2002). Norharmane and harmane are present in tobacco smoke; whereas norharmane inhibits MAO-A and MAO-B with low µM Ki, harmane inhibits MAO-B with much greater potency, having a Ki of 55 nM (Herraiz and Chaparro, 2005). Surprisingly, flavored e-liquids favored by some tobacco smokers as a less harmful nicotine delivery system proved to have significant MAO-A/B inhibitory action, which was attributed to the presence of vanillin (4-hydroxy-3-methoxybenzaldehyde) (Truman, 2019). Indeed, vanillin inhibits MAO-A (IC_50_ 20 µM) and MAO-B (IC_50_ 45 µM), as does eugenol (2-methoxy-4-(prop-2-en-1-yl)phenol), the main odorant of clove oil (Dhiman et al., 2019).

As suggested above, components of tobacco smoke other than β-carbolines may contribute to the net inhibition of MAO in brain of smokers. For example, analysis of cured tobacco leaf revealed the presence of the non-selective MAO inhibitor 2,3,6-trimethyl-1,4-naphthoquinone and the selective MAO-B inhibitor farnesylacetone (Khalil et al., 2006); *in vitro* enzyme competition assays indicated Ki values close to 1 μM, which may or may not be physiologically relevant. A Philip Morris study confirmed the presence of 2,3,6-trimethyl-1,4-naphthoquinone in tobacco or smoke, along with other potential MAO inhibitors such as anabasine, 2-naphthylamine, and farnesylacetone (van der Toorn, 2019).

## PET Imaging of MAO *in vivo*

Molecular imaging by positron emission tomography (PET) affords a window on the living brain, whereby the uptake and binding of selective ligands reveals the molecular targets. We can properly understand PET as a binding assay *in vivo*, where the requirement for external detection by the tomograph calls for integration of certain positron-emitting radionuclides into the tracer molecule of interest. In practice, this is most commonly accomplished using cyclotron-generated fluorine-18 (physical half-life 109 min) or carbon-11 (physical half-life 20 min). The radioactive decay with emission of a positron in the medium of the brain is followed swiftly by annihilation of the positron upon meeting its counterpart electron; the entire energy bound up in the mass of the electron-positron pair (E = mc^2^) then converts into two photons of enormously high energy, approximately 512 keV. These photons, commonly known as gamma rays, fly apart at an angle of just about 180°. If such an emission occurs when an individual’s head is within the aperture of the PET instrument, this counts as “an event”. The accumulating recording of millions of such events over time gives rise to a dynamic PET image, analysis of which maps out the uptake and distribution of binding sites for the tracer molecule. Arguably, MAO-PET was one of the earliest successful uses of this molecular imaging technology to yield new information about human physiology (Fowler, 1987; Fowler, 1988; Kumlien, 1995; Bergström et al., 1997).

In fact, the very first PET study with an MAO ligand employed the neurotoxin MPTP labelled with carbon-11 in the 1-position, i.e., [^11^C]MPTP (Moerlein, 1986). Like MPTP, this radiotracer undergoes oxidative deamination by MAO-B present in astrocytes and some specific neuronal populations, yielding the product [^11^C]-MPP+. That charged metabolite remains inside living cells, thus imparting an increasing PET signal proportional to the local MAO-B activity, as evidenced by the effect of pretreatment with the irreversible MAO inhibitor tranylcypromine, which blocked the specific PET signal in brain of non-human primates. Others confirmed the MAO-B specificity of [^11^C]MPTP in non-human primate brain by blocking its retention through pretreatment with the MAOA/B suicide substrate pargyline, whereas brain trapping was unaffected by pretreatment with the MAO-A-preferring suicide substrate clorgyline (Hartvig, 1986). Other studies in non-human primates with MPTP-induced parkinsonism showed no change in the intensity of [^11^C]-deprenyl uptake in the dopamine depleted striatum, thus indicating that the enzymatic conversion does not take place in the dopamine fibers *per se* (Leenders, 1988). Indeed, it emerged that the toxic MPTP metabolite MPP+ is formed elsewhere (perhaps in astrocytes), whereas it gains access to the vulnerable dopamine neurons via the dopamine uptake site and accumulates to toxic levels via the ionic gradient of living neurons; blockers of dopamine uptake protect against MPTP-induced parkinsonism. Unfortunately, doses of MPTP as low as 1 mg/kg cause an irreversible syndrome of parkinsonism, due to the toxic degeneration of brain dopamine neurons. While the microgram doses of [^11^C]MPTP used in PET studies might be safe, there was a general feeling that better tracers should be developed for human use. Indeed, there soon followed a report on the uptake in mouse and human brain of the MAO-B ligand [^11^C]*N,N*-dimethylphenylethylamine (Shinotoh, 1987). Its uptake in mouse brain was blocked by *L*-deprenyl but not clorgyline pretreatment, and the PET tracer showed abundant accumulation in human brain, especially in the thalamus; the mechanism underlying its trapping in living brain seems to entail enzymatic conversion, as was the case with [^11^C]MPTP.

Initial PET studies in human volunteers showed abundant cerebral uptake of *L*-[^11^C]deprenyl, an MAO-B-preferring suicide substrate, which was blocked by pretreatment with non-radioactive *L*-deprenyl (Fowler, 1987). In serial pig PET studies, MAO-B was blocked by treatment with *L*-deprenyl and the time course of the subsequent return of *L*-[^11^C]deprenyl binding in brain was monitored (Oreland, 1990). This approach indicated a half-life for the turnover of MAO-B in living brain to be about 1 week, which was one of the first such estimates for a specific brain protein. Other pharmacological studies measured the uptake of *L*-[^11^C]deprenyl in brain of healthy volunteers, in conjunction with various doses of the then novel reversible MAO-B inhibitor, Ro 19–6,327 (Bench, 1991; Lammertsma, 1991). Kinetic analyses of the scans with various doses indicated an ID_50_ of 0.3 mg/kg, i.e. the dose of Ro 19–6,327 sufficient to block MAO-B activity in brain by 50%. This competitive inhibition application has since emerged as an important tool in medicinal chemistry for establishing appropriate dose regimens, for example with respect to the necessary frequency of dosing with Ro 19–6,327 required to maintain a constant blockade of MAO-B (Fowler, 1993). The quantitation of *L*-[^11^C]deprenyl uptake in brain is a bit problematic, as it binds so rapidly and irreversibly to brain MAO-B that its uptake is partially limited by cerebral perfusion rate; the greater the blood flow, the greater the PET signal. Aiming to resolve this, the pioneering researchers at Brookhaven National Laboratory and the Karolinska Institute modified *L*-[^11^C]deprenyl by the addition of a deuterium (heavy hydrogen) in the molecule (Fowler, 1988). Since breaking the carbon-deuterium bond by MAO-B is disfavored thermodynamically, the rate of irreversible trapping in brain was slowed substantially compared to ordinary *L*-[^11^C]deprenyl, which allowed the physiological separation of MAO-B binding from cerebral blood flow effects (Fowler, 1995). Alternatives exist, for example the highly specific reversible MAO-B inhibitor 5-[4-(benzyloxy)phenyl]-3-(2-cyanoethyl)-1,3,4-oxadiazol-[^11^C]-2 (3 H)-one, which has nM affinity *in vitro* (Bernard, 1996). PET studies in nonhuman primate indicated a good signal for the detection of MAO-B in brain.

Preliminary work showed the promising reversible MAO-A ligand [^11^C]brofaromine to be largely unsuitable for PET studies due to insufficient specific binding (Ametamey, 1996). The irreversible ligand [^11^C]clorgyline binds to MAO-A in living brain, albeit with a non-MAO-A signal present in the cerebral white matter that may impede quantitation (Fowler, 2001). Nonetheless, the PET study with the suicide-substrate [^11^C]clorgyline PET revealed a substantial inhibition of MAO-A in brain and peripheral organs of habitual smokers (Fowler, 1996). That remarkable observation was the first evidence that MAO-inhibition might be an aspect of the psychopharmacology of tobacco addiction. The finding was confirmed in a completely independent PET study with the alternate MAO-A ligand [^11^C]befloxatine (Leroy, 2009). That study used the arguably superior endpoint of binding potential (BP_ND_), showing a mean 60% reduction of MAO-A activity in cerebral cortex of tobacco smokers, versus a 40% reduction in subcortical regions. Deuterium-substituted [^11^C]deprenyl PET revealed a similar reduction of MAO-B availability in brain of smokers (Fowler, 1998). Further to the question of *H. perforatum*, a [^11^C]harmine PET study did not indicate any MAO-A inhibition in brain of volunteers consuming the product at the dose recommended for depression (Sacher, 2011). This may present an example of the Paracelsus dictum; the presence of MAO-inhibitory substances in extracts of *H. perforatum* may not suffice to inhibit the enzyme *in vivo*, unless consumed in enormous quantities.

Smoking one or two cigarettes increased plasma levels not just of the MAO-B inhibitor norharmane, but also increased levels of the MAO-A inhibitor harmane to 20–30 pg/ml (0.1 nM), then declining with a half-life of about 1 h (Rommelspacher, 2002). This low concentration may explain why smoking a single cigarette was without discernible effect on the cerebral binding of deuterated *L*-[^11^C]deprenyl (Fowler, 1999); the harmane dose is simply too low to evoke much in the way of MAO-B inhibition in brain. Furthermore, there is a pool of endogenous norharmane in human blood, which may be derived from metabolism of plasma tryptophan (Fekkes, 2001); it remains to be established if this endogenous pool has any physiological relevance. The broader action of MAO is indicated by its ubiquity in various organs other than the brain; the aforementioned deuterium isotope effect can be used to confirm specificity of [^11^C]deprenyl binding for MAO-B in various organs of the human body, with a rank order of abundance kidneys ≥ heart > lungs = spleen (Fowler, 2002). This ubiquity makes it difficult to assign a singular role of MAO in physiology.

Early work *in vitro* indicated the β-carboline [^11^C]harmine to have good properties for detection of MAO-A sites in tissue specimens (Goller, 1995). PET studies in pigs showed a very abundant distribution of [^11^C]harmine binding throughout brain, which was entirely blocked by pretreatment with pargyline (Jensen, 2006). That experiment aimed to test a specific hypothesis that blockade of MAO-A would potentiate the effect of amphetamine on dopamine release in brain. Insofar as MAO-A activity contributes to the control of brain dopamine concentrations, and given that amphetamine acts by releasing the intracellular dopamine pool and flooding the synapse, we expected that pargyline pretreatment would potentiate the amphetamine-induced displacement of the dopamine D2 receptor ligand [^11^C]raclopride by dopamine. There was no evidence for any such potentiation according to the PET competition paradigm in anesthetized pigs. Very similarly, pretreatment of rats with pargyline did nothing to enhance the displacement of striatal [^11^C]raclopride binding evoked by amphetamine (Pedersen, 2007). This may stand in contrast to clinical investigations as cited above that MAO inhibition is a salient aspect of tobacco addiction (van Amsterdam, 2006). Similarly, a [^11^C]raclopride PET study in depressed smokers versus healthy controls did not indicate any main effect of depression, although tobacco addiction in depressed individuals tended to decrease the amphetamine-evoked dopamine release (Busto, 2009). That result, in accord with the pig and rat PET studies described above, does not support a strong role for the modulation of amphetamine-induced dopamine release by MAO inhibition from tobacco smoke. Others used [^18^F]altanserin PET to measure availability of serotonin 5-HT_2A_ receptors in brain of depressed individuals; treatment with clomipramine, a re-uptake inhibitor likely to increase interstitial serotonin levels, did reduce [^18^F]altanserin binding, but there was no comparison made with healthy controls. Therefore, that study cannot support any claims about altered serotonin release as a biomarker of depression, or indeed any interaction with MAO-inhibition due to smoking (Larisch, 2003).

Further [^11^C]harmine PET studies in humans indicated that acute serotonin depletion decreased MAO-A binding, whereas pharmacological activation of dopamine synthesis had the opposite effect of increasing MAO-A availability (Sacher, 2012). The authors interpreted these results to indicate a dynamic regulation of MAO-A activity serving to accommodate homeostasis, but did not provide a compelling mechanism to account for that phenomenon. Electroconvulsive therapy for depression, which causes massive serotonin and dopamine release in brain, had only a slight effect in reducing [^11^C]harmine binding (Baldinger-Melich, 2019), which might argue against homeostatic control of MAO-A activity in brain. On the other hand, remission from major depression in patients treated with SSRI medications was not associated with any decline in the elevated [^11^C]harmine binding, suggesting persistence of a trait that might tend to deplete cerebral levels of serotonin and other substances.

[^18^F]Fluoroethylharmol was developed as an alternative to [^11^C]harmine, which presents logistic difficulties due to its very brief physical half-life (Maschauer, 2015). Using a synthesis of findings *in vivo* and *in vitro*, [^18^F]fluoroethylharmol binding results in rat brain suggested a dopamine concentration of 0.4 μM in the striatal compartment containing MAO-A, which seems insufficient to reduce the ligand binding by competition. This might support the implication of the previously described finding (Sacher, 2012) that endogenous MAO substrates do not have directly competitive effects on the activity of MAO in living brain. On the other hand, sevoflurane anesthesia, which is not a direct inhibitor of MAO, decreased deuterated *L*-[^11^C]deprenyl uptake in non-human primate brain by 80%, which is certainly consistent with some novel regulation of MAO-B availability (Varnäs, 2021). Hormones may also play a role here; testosterone treatment for gender dysphoria slightly decreased (−10%) the [^11^C]harmine binding in human brain (Kranz, 2021).

Returning to the theme of [^11^C]harmine PET, studies in nonsmoking patients with major depression indicated a mean 34% greater distribution volume in brain compared to non-depressed controls; this increase was present throughout cerebral cortex, hippocampus, and in subcortical structures (Meyer, 2006). Examination of the scatter plots in Figure 2 of that paper indicated a large effect size (Cohen’s d > 1). While an impressive finding for biological psychiatry, the increased MAO-A availability was not pathognomonic of depression, as there was still considerable overlap in [^11^C]harmine uptake between the two groups, even in the regions of highest binding, i.e., thalamus and cingulate cortex. One might presume that increased MAO-A levels in depressed individuals would predict decreased brain levels of its preferred substrate, i.e., serotonin. This scenario might match with the widely held consideration that major depression is in some sense a serotonin deficiency syndrome, as suggested by the supposed antidepressant efficacy of SSRIs. Furthermore, decreased tonic serotonin levels might alter phasic serotonergic signaling, and could be a factor the meta-analytic finding of slightly (10%) decreased levels of serotonin transporters in brain of depressed individuals compared to age-matched controls (Gryglewski, 2014).

## The Physiological Relevance of MAO Inhibition; the Case of Ayahuasca

We have focused in the preceding section on the molecular imaging of MAO, and its possible relation to neurotransmission and behavior. The physiological relevance of partial MAO-inhibition is a key consideration. Therapy with MAO inhibitors usually aims to obtain nearly complete inhibition of MAO in brain (and consequently in peripheral organs). How important, then, is the transient and partial MAO inhibition seen in the context of tobacco smoking? More dramatically, harmine or other β-carboline alkaloids potentiate the hallucinogenic action of DMT in the context of ayahuasca, presumably by a peripheral action of blocking DMT metabolism in the gut. However, central MAO inhibition might be an independent aspect of the psychopharmacology of harmine in ayahuasca mixtures. Unfortunately, there is scant information about the pharmacokinetics of harmine. An early study followed the disposition of harmine and its metabolites following intravenous injection in humans (0.5 mg/kg) and rats (5 mg/kg) (Slotkin et al., 1970). Much as seen in PET studies with [^11^C]harmine at tracer doses (Jensen, 2006), there was a rapid decline in harmine concentration in human blood (Slotkin et al., 1970). The harmine level in human plasma declined to about 4 μg/ml (10 µM) within minutes after injection, remaining around this level for about 1 h, but falling to about 0.8 μg/ml (2 µM) at 4 hrs after intravenous injection. Given the affinity of harmine for MAO-A *in vitro* (circa 1 nM), we can predict substantial MAO-A inhibition throughout the body and lasting several hours after a dose of 0.5 mg/kg harmine.

While *B. caapi* is an essential component of ayahuasca, the importance of DMT and other alkaloids in *P. virdis* is less clear. In an ayahuasca study, a group of 15 male volunteers (mean body weight 75 kg) consumed 150 ml of a decoction containing 30 mg harmaline, 250 mg harmine and 159 mg tetrahydroharmine, as well as 35 mg DMT (Callaway, 1999). Pharmacokinetic analysis indicated peak plasma levels of 90 ng/ml (500 nM) for harmine at 30 min post-ingestion, which declined with a half-life of 116 min. Tetrahydroharmine attained a peak plasma level of 80 ng/ml (400 nM) at 180 min post ingestion and declining with a half-life of 530 min. The peak plasma level of DMT was 12 ng/ml (60 nM) at 120 min post ingestion, and then declining with a half-life of 260 min. A similar study involving oral administration of ayahuasca to healthy volunteers similarly indicated peak plasma levels of DMT (10 ng/ml) harmaline (3 ng/ml) and harmol (12 ng/ml) at about 2 hrs after administration (Riba, 2003). These concentrations had substantially declined within 6 hrs after ingestion, although tetrahydroharmine and harmalol levels tended to persist for up to 24 h. Remarkably, the treatment was without effect on the urine levels of the MAO-A serotonin metabolite 5-hydroxyindoleacetic acid (5-HIAA), nor was there any decrease in levels of catecholamine metabolites despite the transiently high plasma levels of MAO inhibitors. This result might be confounded by the prolonged urine collection period (24 h) relative to duration of the peak plasma levels of some of the β-carbolines. More importantly perhaps, the subjective effects of the ayahuasca peaked at about 2 hrs after ingestion, and had declined almost to baseline 4 hrs later. Thus, there is a close alignment of the plasma concentrations of DMT and some β-carbolines with the intensity of the psychotropic effects. Brito-da-Costa et al. (2020) have recently presented a detailed review of the literature on DMT and β-carboline kinetics presenting a wealth of details. Wang (2019) produced a similarly detailed investigation of the pharmacokinetics of various alkaloids in rats fed with seeds of *P. harmala*, the rue plant mentioned above. A single rather high dose of the seeds (150 mg/kg) resulted in plasma harmaline levels above 10 ng/ml persisting for at least 12 h, whereas harmine levels were around 100 ng/ml for a similar period, but harmol levels were generally below 10 ng/ml. The corresponding concentrations were much lower in rats treated with a low *P. harmala* dose (15 mg/kg), with harmaline and harmine being barely detectable in plasma. This suggests a non-linear dose-response, making it difficult to extrapolate these results to human ingestion of *P. harmala*; there is a case report of fatal toxicity due to *P. harmala* ingestion (Ghizlane, 2021), whereas a study from Morocco indicated a 6% fatality rate in a series of 200 cases of poisoning marked by neurological, gastrointestinal and cardiovascular signs (Achour, 2012). We cannot attribute these effects to β-carbolines *per se*. However, the causal relationship may be clearer in a fatality following consumption of unspecified extracts, with post mortem findings of DMT (20 μg/L; 100 nM) the notorious toad toxin 5-methoxy-*N,N*-dimethyltryptamine (2 mg/L; 10 µM) and harmine (0.2 mg/L; 1 µM) (Sklerov, 2005). However, that case reported was critiqued in the contemporary literature (Callaway, 2006). Severe poisoning by β-carbolines is certainly possible, but seems to be a rare occurrence, and might properly be attributable to interactions with other pharmacologically active substances, as in the case of the “cheese effect” discussed above.

## Conclusion

In summary, β-carbolines and other classes of MAO inhibitors are present in tobacco and many other plants, and likely contribute to the psychopharmacology of tobacco usage and perhaps dependence. However, there is incomplete documentation of the dose-response relationships; a low degree of MAO inhibition may potentiate the reinforcing properties of nicotine or amphetamine, but complete MAO blockade may be aversive. There is a particular involvement of MAO-A inhibition in the pharmacology of ayahuasca, which is generally attributed to its potentiation of the bioavailability of the actively hallucinogenic ingredient DMT. However, not all ayahuasca preparations contain significant amounts of DMT (McKenna 1984a; Callaway et al., 2005), and some Indigenous groups of the Amazon employ *B. caapi* without admixture plants, in the context of healing and initiatory rites (Rodd, 2008; Politi et al., 2021); we need to consider better the polypharmacolgy of ayahuasca and the possible independent effects of β-carbolines on brain function. The β-carbolines present in *B. caapi* were found for instance to stimulate neurogenesis in adult mice (Morales-García, 2017) and harmane and norharmane (i.e., the alkaloids present in tobacco) were associated with antidepressant-like effects in the mouse forced swim test (Farzin and Mansouri, 2006). Furthermore, it remains unknown if MAO inhibition increases the brain concentration of endogenous DMT, as is well established for the classical trace amines. In the context of tobacco use, what accounts for the composite effect on MAO-activity? Self-rolled cigarettes produce particulate with higher MAO-inhibitory activity than is the case in tailor-made cigarettes (Lewis, 2012). Is this due to the variety of tobacco species used, or its curing process? What is the alkaloid content of Indian beedis and do the wrapping leaves (*Diospyros melanoxylon*) contain pharmacologically active compounds? Clove cigarettes (kreteks), which are popular in Indonesia, contain half as much nicotine as typical cigarettes, but their aromatic quality may encourage deeper inhalation and thus greater absorption of alkaloids (Malson, 2003). Where there is smoke, there is fire; this aphorism does not apply to snuff and chewing tobacco, which have not been addressed in this brief contribution and are scantly investigated with respect to their psychopharmacology. The composition of MAO-inhibiting alkaloids in different tobacco species also remains unknown. *Nicotiana rustica*, for instance (“Aztec tobacco,” in Peru known as “Mapacho”) in the Amazon region is considered an important medicinal plant and is a frequent component of Amerindian ethnobotanical mixtures, also elsewhere. In traditional Amazonian medicine, this tobacco species is prepared as a liquid remedy and is ingested orally to treat mental health and other ailments (Berlowitz, 2020); the rapid first pass clearance of oral nicotine could mean that unidentified alkaloids other than nicotine play a role in the psychopharmacology of this orally taken ethnomedical application of *N. rustica*.

In this account, we have emphasized the family resemblance between tobacco and ayahuasca; both substances contain a mixture of alkaloids, with considerable potential for interaction, especially through the inhibition of MAO by β-carbolines and other competitors. We may inadvertently have raised more questions than we have answered, but we see a clear need for a more systematic examination of the separate and combined contributions of polypharmacy in the contexts or tobacco or ayahuasca consumption.

## Footnotes

1 Harmane/norharmane are often also written as harman/norharman; we follow the former convention in this manuscript.

## References

[B1] Achour S. (2012). Peganum Harmala L. Poisoning in Morocco: about 200 Cases. Therapie 67 (1), 53–58. 10.2515/therapie/2012003 22487503

[B2] Ametamey S. M. (1996). Radiosynthesis of [11C]brofaromine, a Potential Tracer for Imaging Monoamine Oxidase A. Nucl. Med. Biol. 23 (3), 229–234. 10.1016/0969-8051(95)02051-9 8782230

[B3] Baldinger-Melich P. (2019). The Effect of Electroconvulsive Therapy on Cerebral Monoamine Oxidase A Expression in Treatment-Resistant Depression Investigated Using Positron Emission Tomography. Brain Stimul 12 (3), 714–723. 10.1016/j.brs.2018.12.976 30635228

[B4] Bench C. J. (1991). Measurement of Human Cerebral Monoamine Oxidase Type B (MAO-B) Activity with Positron Emission Tomography (PET): a Dose Ranging Study with the Reversible Inhibitor Ro 19-6327. Eur. J. Clin. Pharmacol. 40 (2), 169–173. 10.1007/BF00280072 1906004

[B5] Bergström M. Westerberg G. Långström B. (1997). 11C-harmine as a Tracer for Monoamine Oxidase A (MAO-A): *In Vitro* and *In Vivo* Studies. Nucl. Med. Biol. 24 (4), 287 9257326 10.1016/s0969-8051(97)00013-9

[B6] Berlowitz I. (2020). Tobacco Is The Chief Medicinal Plant In My Work”: Therapeutic Uses Of Tobacco In Peruvian Amazonian Medicine Exemplified By the Work Of a Maestro Tabaquero. Front. Pharmacol. 11. 10.3389/fphar.2020.594591 PMC7576958 33117182

[B7] Bernard S. (1996). Synthesis and *In Vivo* Studies of a Specific Monoamine Oxidase B Inhibitor: 5-[4-(benzyloxy)phenyl]-3-(2-Cyanoethyl)- 1,3,4-Oxadiazol-[11c]-2(3h)-One. Eur. J. Nucl. Med. 23 (2), 150–156. 10.1007/BF01731838 8925849

[B8] Bladt S. Wagner H. (1994). Inhibition of MAO by Fractions and Constituents of hypericum Extract. J. Geriatr. Psychiatry Neurol. 7 (Suppl. 1), S57–S59. 10.1177/089198879400700115 7857511

[B9] Blaha C. D. Coury A. Phillips A. G. (1996). Does Monoamine Oxidase Inhibition by Pargyline Increase Extracellular Dopamine Concentrations in the Striatum? Neuroscience 75 (2), 543–550. 10.1016/0306-4522(96)00289-8 8931017

[B10] Bortolato M., (2013). Monoamine Oxidase A and A/B Knockout Mice Display Autistic-like Features. Int. J. Neuropsychopharmacol. 16 (4), 869–888. 10.1017/S1461145712000715 22850464 PMC3517692

[B11] Breyer-Pfaff U., (1996). Elevated Norharman Plasma Levels in Alcoholic Patients and Controls Resulting from Tobacco Smoking. Life Sci. 58 (17), 1425–1432. 10.1016/0024-3205(96)00112-9 8622568

[B12] Brito-da-Costa A. M., (2020). Toxicokinetics and Toxicodynamics of Ayahuasca Alkaloids N,N-Dimethyltryptamine (DMT), Harmine, Harmaline and Tetrahydroharmine: Clinical and Forensic Impact. Pharmaceuticals (Basel) 13 (11). 10.3390/ph13110334 PMC7690791 33114119

[B13] Brown E. E., (1991). Interstitial 3-methoxytyramine Reflects Striatal Dopamine Release: an *In Vivo* Microdialysis Study. J. Neurochem. 57 (2), 701–707. 10.1111/j.1471-4159.1991.tb03802.x 1906527

[B14] Brunner H. G. (1993). Abnormal Behavior Associated with a point Mutation in the Structural Gene for Monoamine Oxidase A. Science 262 (5133), 578–580. 10.1126/science.8211186 8211186

[B15] Busto U. E. (2009). Dopaminergic Activity in Depressed Smokers: a Positron Emission Tomography Study. Synapse 63 (8), 681–689. 10.1002/syn.20646 19360907 PMC2761223

[B16] Buu N. T. Lussier C. (1989). Consequences of Monoamine Oxidase Inhibition: Increased Vesicular Accumulation of Dopamine and Norepinephrine and Increased Metabolism by Catechol-O-Methyltransferase and Phenolsulfotransferase. Prog. Neuropsychopharmacol. Biol. Psychiatry 13 (3-4), 563–568. 10.1016/0278-5846(89)90147-4 2748880

[B17] Callaway J. C. (2006). A Demand for Clarity Regarding a Case Report on the Ingestion of 5-Methoxy-N, N-Dimethyltryptamine (5-MeO-DMT) in an Ayahuasca Preparation. J. Anal. Toxicol. 30 (6), 406–407. 10.1093/jat/30.6.406 16872575

[B18] Callaway J. C. Brito G. S. Neves E. S. (2005). Phytochemical Analyses of Banisteriopsis Caapi and Psychotria Viridis. J. Psychoactive Drugs 37 (2), 145–150. 10.1080/02791072.2005.10399795 16149327

[B19] Callaway J. C. (1999). Pharmacokinetics of Hoasca Alkaloids in Healthy Humans. J. Ethnopharmacol 65 (3), 243–256. 10.1016/s0378-8741(98)00168-8 10404423

[B20] Carbonaro T. M. Gatch M. B. (2016). Neuropharmacology of N,N-dimethyltryptamine. Brain Res. Bull. 126 (Pt 1), 74–88. 10.1016/j.brainresbull.2016.04.016 27126737 PMC5048497

[B21] Castagnoli K. Murugesan T. (2004). Tobacco Leaf, Smoke and Smoking, MAO Inhibitors, Parkinson's Disease and Neuroprotection; Are There Links? Neurotoxicology 25 (1-2), 279–291. 10.1016/S0161-813X(03)00107-4 14697903

[B22] Cumming P., (1992). Formation and Clearance of Interstitial Metabolites of Dopamine and Serotonin in the Rat Striatum: an *In Vivo* Microdialysis Study. J. Neurochem. 59 (5), 1905–1914. 10.1111/j.1471-4159.1992.tb11026.x 1383428

[B23] Cumming P., (2021). Molecular and Functional Imaging Studies of Psychedelic Drug Action in Animals and Humans. Molecules 26 (9). 10.3390/molecules26092451 PMC8122807 33922330

[B24] Dhiman P. Malik N. Khatkar A. (2019). Lead Optimization for Promising Monoamine Oxidase Inhibitor from Eugenol for the Treatment of Neurological Disorder: Synthesis and In Silico Based Study. BMC Chem. 13 (1), 38. 10.1186/s13065-019-0552-4 31384786 PMC6661809

[B25] Durden D. A. Philips S. R. (1980). Kinetic Measurements of the Turnover Rates of Phenylethylamine and Tryptamine *In Vivo* in the Rat Brain. J. Neurochem. 34 (6), 1725–1732. 10.1111/j.1471-4159.1980.tb11267.x 7381498

[B26] Farzin D. Mansouri N. (2006). Antidepressant-like Effect of Harmane and Other β-carbolines in the Mouse Forced Swim Test. Eur. Neuropsychopharmacol. 16 (5), 324–328. 10.1016/j.euroneuro.2005.08.005 16183262

[B27] Fekkes D., (2001). Tryptophan: a Precursor for the Endogenous Synthesis of Norharman in Man. Neurosci. Lett. 303 (3), 145–148. 10.1016/s0304-3940(01)01750-5 11323106

[B28] Fornai F., (1999). Striatal Dopamine Metabolism in Monoamine Oxidase B-Deficient Mice: a Brain Dialysis Study. J. Neurochem. 73 (6), 2434–2440. 10.1046/j.1471-4159.1999.0732434.x 10582603

[B29] Fowler J. S., (1996). Brain Monoamine Oxidase A Inhibition in Cigarette Smokers. Proc. Natl. Acad. Sci. U S A. 93 (24), 14065–14069. 10.1073/pnas.93.24.14065 8943061 PMC19495

[B30] Fowler J. S., (1987). Mapping Human Brain Monoamine Oxidase A and B with 11C-Labeled Suicide Inactivators and PET. Science 235 (4787), 481–485. 10.1126/science.3099392 3099392

[B31] Fowler J. S., (1988). Mechanistic Positron Emission Tomography Studies: Demonstration of a Deuterium Isotope Effect in the Monoamine Oxidase-Catalyzed Binding of [11C]L-Deprenyl in Living Baboon Brain. J. Neurochem. 51 (5), 1524–1534. 10.1111/j.1471-4159.1988.tb01121.x 3139834

[B32] Fowler J. S., (1993). Monoamine Oxidase B (MAO B) Inhibitor Therapy in Parkinson's Disease: the Degree and Reversibility of Human Brain MAO B Inhibition by Ro 19 6327. Neurology 43 (10), 1984–1992. 10.1212/wnl.43.10.1984 8413955

[B33] Fowler J. S., (1998). Neuropharmacological Actions of Cigarette Smoke: Brain Monoamine Oxidase B (MAO B) Inhibition. J. Addict. Dis. 17 (1), 23–34. 10.1300/J069v17n01_03 9549600

[B34] Fowler J. S., (2001). Non-MAO A Binding of Clorgyline in white Matter in Human Brain. J. Neurochem. 79 (5), 1039–1046. 10.1046/j.1471-4159.2001.00649.x 11739617

[B35] Fowler J. S. (2002). PET Imaging of Monoamine Oxidase B in Peripheral Organs in Humans. J. Nucl. Med. 43 (10), 1331 12368371

[B36] Fowler J. S. (1995). Selective Reduction of Radiotracer Trapping by Deuterium Substitution: Comparison of Carbon-11-L-Deprenyl and Carbon-11-Deprenyl-D2 for MAO B Mapping. J. Nucl. Med. 36 (7), 1255 7790952

[B37] Fowler J. S. (1999). Smoking a Single Cigarette Does Not Produce a Measurable Reduction in Brain MAO B in Non-smokers. Nicotine Tob. Res. 1 (4), 325–329. 10.1080/14622299050011451 11072429

[B38] Gainetdinov R. R. Hoener M. C. Berry M. D. (2018). Trace Amines and Their Receptors. Pharmacol. Rev. 70 (3), 549–620. 10.1124/pr.117.015305 29941461

[B39] Ghizlane E. A. (2021). Fatal Poisoning of Pregnant Women by Peganum Harmala L.: A Case Reports. Ann. Med. Surg. (Lond) 68, 102649. 10.1016/j.amsu.2021.102649 34401132 PMC8350184

[B40] Gillman P. K. (2011). Advances Pertaining to the Pharmacology and Interactions of Irreversible Nonselective Monoamine Oxidase Inhibitors. J. Clin. Psychopharmacol. 31 (1), 66–74. 10.1097/JCP.0b013e31820469ea 21192146

[B41] Godar S. C. (2014). The Aggression and Behavioral Abnormalities Associated with Monoamine Oxidase A Deficiency Are Rescued by Acute Inhibition of Serotonin Reuptake. J. Psychiatr. Res. 56, 1–9. 10.1016/j.jpsychires.2014.04.014 24882701 PMC4114985

[B42] Goller L. (1995). Mao-a Enzyme Binding in Bladder-Cancer Characterized with [C-11] Harmine in Frozen-Section Autoradiography. Oncol. Rep. 2 (5), 717–721. 10.3892/or.2.5.717 21597803

[B43] Gryglewski G. (2014). Meta-analysis of Molecular Imaging of Serotonin Transporters in Major Depression. J. Cereb. Blood Flow Metab. 34 (7), 1096–1103. 10.1038/jcbfm.2014.82 24802331 PMC4083395

[B44] Guillem K. (2006). Monoamine Oxidase A rather Than Monoamine Oxidase B Inhibition Increases Nicotine Reinforcement in Rats. Eur. J. Neurosci. 24 (12), 3532–3540. 10.1111/j.1460-9568.2006.05217.x 17229101

[B45] Harris A. C. Muelken P. LeSage M. G. (2020). β-Carbolines Found in Cigarette Smoke Elevate Intracranial Self-Stimulation Thresholds in Rats. Pharmacol. Biochem. Behav. 198, 173041. 10.1016/j.pbb.2020.173041 32926882 PMC7554228

[B46] Hartvig P. (1986). Influence of Monoamine Oxidase Inhibitors and a Dopamine Uptake Blocker on the Distribution of 11C-N-Methyl-4-Phenyl-1,2,3,6-Tetrahydropyridine, 11C-MPTP, in the Head of the Rhesus Monkey. Acta Neurol. Scand. 74 (1), 10–16. 10.1111/j.1600-0404.1986.tb04618.x 3490110

[B47] Herraiz T. Chaparro C. (2005). Human Monoamine Oxidase Is Inhibited by Tobacco Smoke: Beta-Carboline Alkaloids Act as Potent and Reversible Inhibitors. Biochem. Biophys. Res. Commun. 326 (2), 378–386. 10.1016/j.bbrc.2004.11.033 15582589

[B48] Herraiz T. Guillén H. (2018). Monoamine Oxidase-A Inhibition and Associated Antioxidant Activity in Plant Extracts with Potential Antidepressant Actions. Biomed. Res. Int. 2018, 4810394. 10.1155/2018/4810394 29568754 PMC5820675

[B49] Herraiz T. (2004). Relative Exposure to Beta-Carbolines Norharman and Harman from Foods and Tobacco Smoke. Food Addit Contam. 21 (11), 1041–1050. 10.1080/02652030400019844 15764332

[B50] Janiger O. de Rios M. D. (1973). Suggestive Hallucinogenic Properties of Tobacco. Med. Anthropol. Newsl. 4 (4), 6–11. 10.1525/maq.1973.4.4.02a00050

[B51] Jensen S. B. (2006). Effect of Monoamine Oxidase Inhibition on Amphetamine-Evoked Changes in Dopamine Receptor Availability in the Living Pig: a Dual Tracer PET Study with [11C]harmine and [11C]raclopride. Synapse 59 (7), 427–434. 10.1002/syn.20258 16485265

[B52] Jeong G. S. (2021). Selective Inhibition of Human Monoamine Oxidase B by 5-Hydroxy-2-Methyl-Chroman-4-One Isolated from an Endogenous Lichen Fungus Daldinia Fissa. J. Fungi (Basel) 7 (2). 10.3390/jof7020084 PMC7911959 33530616

[B53] Khalil A. A. Davies B. Castagnoli N. Jr. (2006). Isolation and Characterization of a Monoamine Oxidase B Selective Inhibitor from Tobacco Smoke. Bioorg. Med. Chem. 14 (10), 3392–3398. 10.1016/j.bmc.2005.12.057 16458520

[B54] Kitanaka N. (2010). Low-dose Pretreatment with Clorgyline Decreases the Levels of 3-Methoxy-4-Hydroxyphenylglycol in the Striatum and Nucleus Accumbens and Attenuates Methamphetamine-Induced Conditioned Place Preference in Rats. Neuroscience 165 (4), 1370–1376. 10.1016/j.neuroscience.2009.11.058 19958817

[B55] Kitanaka N. (2006). Methamphetamine Reward in Mice as Assessed by Conditioned Place Preference Test with Supermex Sensors: Effect of Subchronic Clorgyline Pretreatment. Neurochem. Res. 31 (6), 805–813. 10.1007/s11064-006-9081-3 16791472

[B56] Koyiparambath V. P. (2021). Deciphering the Detailed Structure-Activity Relationship of Coumarins as Monoamine Oxidase Enzyme Inhibitors-An Updated Review. Chem. Biol. Drug Des. 98 (4), 655–673. 10.1111/cbdd.13919 34233082

[B57] Kranz G. S., (2021). High-dose Testosterone Treatment Reduces Monoamine Oxidase A Levels in the Human Brain: A Preliminary Report. Psychoneuroendocrinology 133, 105381. 10.1016/j.psyneuen.2021.105381 34416504

[B58] Kumlien E. (1995). Positron Emission Tomography with [11C]deuterium-Deprenyl in Temporal Lobe Epilepsy. Epilepsia 36 (7), 712–721. 10.1111/j.1528-1157.1995.tb01051.x 7555990

[B59] Lammertsma A. A. (1991). Measurement of Cerebral Monoamine Oxidase B Activity Using L-[11C]deprenyl and Dynamic Positron Emission Tomography. J. Cereb. Blood Flow Metab. 11 (4), 545–556. 10.1038/jcbfm.1991.103 1904879

[B60] Larisch R. (2003). Influence of Synaptic Serotonin Level on [18F]altanserin Binding to 5HT2 Receptors in Man. Behav. Brain Res. 139 (1-2), 21–29. 10.1016/s0166-4328(01)00412-0 12642173

[B61] Leenders K. L. (1988). Unilateral MPTP Lesion in a Rhesus Monkey: Effects on the Striatal Dopaminergic System Measured *In Vivo* with PET Using Various Novel Tracers. Brain Res. 445 (1), 61–67. 10.1016/0006-8993(88)91074-8 3259152

[B62] Leroy C. (2009). Cerebral Monoamine Oxidase A Inhibition in Tobacco Smokers Confirmed with PET and [11C]befloxatone. J. Clin. Psychopharmacol. 29 (1), 86–88. 10.1097/JCP.0b013e31819e98f 19142115

[B63] Lewis A. J. (2012). Monoamine Oxidase Inhibitory Activity in Tobacco Smoke Varies with Tobacco Type. Tob. Control. 21 (1), 39–43. 10.1136/tc.2010.040287 21636610

[B64] Li S. (2014). Sex Differences in Yohimbine-Induced Increases in the Reinforcing Efficacy of Nicotine in Adolescent Rats. Addict. Biol. 19 (2), 156–164. 10.1111/j.1369-1600.2012.00473.x 22784103

[B65] Liu W. (2021). Degradation of β-Carbolines Harman and Norharman in Edible Oils during Heating. Molecules 26 (22), 7018. 10.3390/molecules26227018 34834111 PMC8623535

[B66] Ma L., (2020). Purpurin Exerted Antidepressant-like Effects on Behavior and Stress axis Reactivity: Evidence of Serotonergic Engagement. Psychopharmacology (Berl) 237 (3), 887–899. 10.1007/s00213-019-05422-w 31900524

[B67] Malson J. L. (2003). Clove Cigarette Smoking: Biochemical, Physiological, and Subjective Effects. Pharmacol. Biochem. Behav. 74 (3), 739–745. 10.1016/s0091-3057(02)01076-6 12543240

[B68] Mappin-Kasirer B., (2020). Tobacco Smoking and the Risk of Parkinson Disease: A 65-year Follow-Up of 30,000 Male British Doctors. Neurology 94 (20), e2132–e2138. 10.1212/WNL.0000000000009437 32371450 PMC7526668

[B69] Maschauer S. (2015). Specific Binding of [(18)F]fluoroethyl-Harmol to Monoamine Oxidase A in Rat Brain Cryostat Sections, and Compartmental Analysis of Binding in Living Brain. J. Neurochem. 135 (5), 908–917. 10.1111/jnc.13370 26386360

[B70] McKenna D. J. (1984). Monoamine oxidase inhibitors South Am. hallucinogenic plants: tryptamine beta-carboline constituents ayahuasca. J Ethnopharmacol 10 (2), 195–223. 10.1016/0378-8741(84)90003-5 6587171

[B71] McKenna D. J. (1984b). Monoamine oxidase inhibitors South Am. hallucinogenic plants Part 2: Constituents orally-active Myristicaceous hallucinogens. J Ethnopharmacol 12 (2), 179–211. 10.1016/0378-8741(84)90048-5 6521493

[B72] Meyer J. H. (2006). Elevated Monoamine Oxidase a Levels in the Brain: an Explanation for the Monoamine Imbalance of Major Depression. Arch. Gen. Psychiatry 63 (11), 1209–1216. 10.1001/archpsyc.63.11.1209 17088501

[B73] Moerlein S. M. (1986). Regional Cerebral Pharmacokinetics of the Dopaminergic Neurotoxin 1-Methyl-4-Phenyl-1,2,3,6-Tetrahydropyridine as Examined by Positron Emission Tomography in a Baboon Is Altered by Tranylcypromine. Neurosci. Lett. 66 (2), 205–209. 10.1016/0304-3940(86)90191-6 3487753

[B74] Moloudizargari M., (2013). Pharmacological and Therapeutic Effects of Peganum Harmala and its Main Alkaloids. Pharmacogn Rev. 7 (14), 199–212. 10.4103/0973-7847.120524 24347928 PMC3841998

[B75] Morales-García J. A., (2017). The Alkaloids of Banisteriopsis Caapi, the Plant Source of the Amazonian Hallucinogen Ayahuasca, Stimulate Adult Neurogenesis *In Vitro* . Scientific Rep. 7 (1), 5309. 10.1038/s41598-017-05407-9 PMC5509699 28706205

[B76] Murphy D. L., (1990). Marked Amine and Amine Metabolite Changes in Norrie Disease Patients with an X-Chromosomal Deletion Affecting Monoamine Oxidase. J. Neurochem. 54 (1), 242–247. 10.1111/j.1471-4159.1990.tb13307.x 2293615

[B77] Ona G. S. Dos Santos R. G. Hallak J. E. C. Bouso J. C. (2020). Polypharmacology Or “Pharmacological Promiscuity” in Psychedelic Research: What Are We Missing? ACS Chem. Neurosci. 11 (20), 3191–3193. 10.1021/acschemneuro.0c00614 33021777

[B78] Oreland L., (1990). Turnover of Monoamine Oxidase B (MAO-B) in Pig Brain by Positron Emission Tomography Using 11C-L-Deprenyl. J. Neural Transm. Suppl. 32, 55–59. 10.1007/978-3-7091-9113-2_6 2128512

[B79] Ott J. (1999). Pharmahuasca: Human Pharmacology of Oral DMT Plus Harmine. J. Psychoactive Drugs 31 (2), 171–177. 10.1080/02791072.1999.10471741 10438001

[B80] Pedersen K., (2007). Mapping the Amphetamine-Evoked Changes in [11C]raclopride Binding in Living Rat Using Small Animal PET: Modulation by MAO-Inhibition. Neuroimage 35 (1), 38–46. 10.1016/j.neuroimage.2006.11.038 17223363

[B81] Poindexter E. H. Carpenter R. D. (1962). The Isolation of Harmane and Norharmane from Tobacco and Cigarette Smoke. Phytochemistry 1 (3), 215–221. 10.1016/s0031-9422(00)82825-3

[B82] Politi M. Friso F. Saucedo G. Torres J. (2021). Traditional Use of Banisteriopsis Caapi Alone and its Application in a Context of Drug Addiction Therapy. J. Psychoactive Drugs 53 (1), 76–84. 10.1080/02791072.2020.1820641 32985365

[B83] Quitkin F. Rifkin A. Klein D. F. (1979). Monoamine Oxidase Inhibitors. A Review of Antidepressant Effectiveness. Arch. Gen. Psychiatry 36 (7), 749–760. 10.1001/archpsyc.1979.01780070027003 454092

[B84] Ramadan S. (2021). Dismantling Parkinson's Disease with Herbs: MAO-B Inhibitory Activity and Quantification of Chemical Constituents Using HPLC-MS/MS of Egyptian Local Market Plants. Nat. Prod. Res., 1–6. 10.1080/14786419.2021.2013836 34894897

[B85] Riba J. (2003). Human Pharmacology of Ayahuasca: Subjective and Cardiovascular Effects, Monoamine Metabolite Excretion, and Pharmacokinetics. J. Pharmacol. Exp. Ther. 306 (1), 73–83. 10.1124/jpet.103.049882 12660312

[B86] Rodd R. (2008). Reassessing the Cultural and Psychopharmacological Significance of Banisteriopsis Caapi: Preparation, Classification and Use Among the Piaroa of Southern Venezuela. J. Psychoactive Drugs 40 (3), 301–307. 10.1080/02791072.2008.10400645 19004422

[B87] Rommelspacher H., (2002). The Levels of Norharman Are High Enough after Smoking to Affect Monoamineoxidase B in Platelets. Eur. J. Pharmacol. 441 (1-2), 115–125. 10.1016/s0014-2999(02)01452-8 12007928

[B88] Sacher J. (2012). Dynamic, Adaptive Changes in MAO-A Binding after Alterations in Substrate Availability: an *In Vivo* [(11)C]-harmine Positron Emission Tomography Study. J. Cereb. Blood Flow Metab. 32 (3), 443–446. 10.1038/jcbfm.2011.184 22186668 PMC3293124

[B89] Sacher J. (2011). Monoamine Oxidase A Inhibitor Occupancy during Treatment of Major Depressive Episodes with Moclobemide or St. John's Wort: an [11C]-Harmine PET Study. J. Psychiatry Neurosci. 36 (6), 375–382. 10.1503/jpn.100117 21463543 PMC3201991

[B90] Schoepp D. D. Azzaro A. J. (1981). Specificity of Endogenous Substrates for Types A and B Monoamine Oxidase in Rat Striatum. J. Neurochem. 36 (6), 2025–2031. 10.1111/j.1471-4159.1981.tb10829.x 6787175

[B91] Shih J. C. Chen K. (2004). Regulation of MAO-A and MAO-B Gene Expression. Curr. Med. Chem. 11 (15), 1995–2005. 10.2174/0929867043364757 15279563

[B92] Shih J. C. Chen K. (1999). MAO-A and -B Gene Knock-Out Mice Exhibit Distinctly Different Behavior. Neurobiology (Bp) 7 (2), 235. 10591056

[B93] Shih J. C. Chen K. Ridd M. J. (1999). Monoamine Oxidase: from Genes to Behavior. Annu. Rev. Neurosci. 22, 197–217. 10.1146/annurev.neuro.22.1.197 10202537 PMC2844879

[B94] Shinotoh H. (1987). Kinetics of [11C]N,N-dimethylphenylethylamine in Mice and Humans: Potential for Measurement of Brain MAO-B Activity. J. Nucl. Med. 28 (6), 1006. 3495646

[B95] Shulman K. I. Herrmann N. Walker S. E. (2013). Current Place of Monoamine Oxidase Inhibitors in the Treatment of Depression. CNS Drugs 27 (10), 789–797. 10.1007/s40263-013-0097-3 23934742

[B96] Simão A. Y. (2019). Toxicological Aspects and Determination of the Main Components of Ayahuasca: A Critical Review. Medicines (Basel) 6 (4). 10.3390/medicines6040106 PMC6963515 31635364

[B97] Sklerov J. (2005). A Fatal Intoxication Following the Ingestion of 5-Methoxy-N,n-Dimethyltryptamine in an Ayahuasca Preparation. J. Anal. Toxicol. 29 (8), 838–841. 10.1093/jat/29.8.838 16356341

[B98] Slotkin T. A. DiStefano V. Au W. Y. (1970). Blood Levels and Urinary Excretion of Harmine and its Metabolites in Man and Rats. J. Pharmacol. Exp. Ther. 173 (1), 26–30. 5442301

[B99] Smith T. T. (2015). Effects of MAO Inhibition and a Combination of Minor Alkaloids, β-carbolines, and Acetaldehyde on Nicotine Self-Administration in Adult Male Rats. Drug Alcohol Depend 155, 243–252. 10.1016/j.drugalcdep.2015.07.002 26257022 PMC4581969

[B100] Spijkerman R. (2002). The Impact of Smoking and Drinking on Plasma Levels of Norharman. Eur. Neuropsychopharmacol. 12 (1), 61–71. 10.1016/s0924-977x(01)00143-2 11788242

[B101] Suzuki O. Oya M. Katsumata Y. (1979). Oxidation of P-, M- and O-Tyramine by Type A and Type B Monoamine Oxidase. Biochem. Pharmacol. 28 (17), 2682–2684. 10.1016/0006-2952(79)90050-9 518681

[B102] Tarkowská D. (2020). A Fast and Reliable UHPLC-MS/MS-Based Method for Screening Selected Pharmacologically Significant Natural Plant Indole Alkaloids. Molecules 25 (14), 3274. 10.3390/molecules25143274 PMC7397342 32708364

[B103] Thase M. E. Trivedi M. H. Rush A. J. (1995). MAOIs in the Contemporary Treatment of Depression. Neuropsychopharmacology 12 (3), 185–219. 10.1016/0893-133X(94)00058-8 7612154

[B104] Truman P. Grounds P. Brennan K. A. (2017). Monoamine Oxidase Inhibitory Activity in Tobacco Particulate Matter: Are Harman and Norharman the Only Physiologically Relevant Inhibitors? Neurotoxicology 59, 22–26. 10.1016/j.neuro.2016.12.010 28057462

[B105] Truman P. (2019). Monoamine Oxidase Inhibitory Activity of Flavoured E-Cigarette Liquids. Neurotoxicology 75, 123–128. 10.1016/j.neuro.2019.09.010 31536738

[B106] Ulrich S. Ricken R. Adli M. (2017). Tranylcypromine in Mind (Part I): Review of Pharmacology. Eur. Neuropsychopharmacol. 27 (8), 697–713. 10.1016/j.euroneuro.2017.04.003 28655495

[B107] van Amsterdam J., (2006). Contribution of Monoamine Oxidase (MAO) Inhibition to Tobacco and Alcohol Addiction. Life Sci. 79 (21), 1969–1973. 10.1016/j.lfs.2006.06.010 16884739

[B108] Van Den Eijnden R. Spijkerman R. Fekkes D. (2003). Craving for Cigarettes Among Low and High Dependent Smokers: Impact of Norharman. Addict. Biol. 8 (4), 463–472. 10.1080/13556210310001646457 14690883

[B109] van der Toorn M., (2019). Comparison of Monoamine Oxidase Inhibition by Cigarettes and Modified Risk Tobacco Products. Toxicol. Rep. 6, 1206–1215. 10.1016/j.toxrep.2019.11.008 31768332 PMC6872813

[B110] Varnäs K. (2021). Effects of Sevoflurane Anaesthesia on Radioligand Binding to Monoamine Oxidase-B *In Vivo* . Br. J. Anaesth. 126 (1), 238–244. 10.1016/j.bja.2020.08.052 33036760 PMC8258980

[B111] Villégier A. S. (2007). Involvement of Alpha1-Adrenergic Receptors in Tranylcypromine Enhancement of Nicotine Self-Administration in Rat. Psychopharmacology (Berl) 193 (4), 457–465. 10.1007/s00213-007-0799 17486319

[B112] Wang Y. (2019). Subchronic Toxicity and Concomitant Toxicokinetics of Long-Term Oral Administration of Total Alkaloid Extracts from Seeds of Peganum Harmala Linn: A 28-day Study in Rats. J. Ethnopharmacol 238, 111866. 10.1016/j.jep.2019.111866 30970283

[B113] Yang H. Y. Neff N. H. (1973). Beta-phenylethylamine: a Specific Substrate for Type B Monoamine Oxidase of Brain. J. Pharmacol. Exp. Ther. 187 (2), 365–371. 4748552

[B114] Youdim M. B. Weinstock M. (2004). Therapeutic Applications of Selective and Non-selective Inhibitors of Monoamine Oxidase A and B that Do Not Cause Significant Tyramine Potentiation. Neurotoxicology 25 (1-2), 243–250. 10.1016/S0161-813X(03)00103-7 14697899

[B115] Zhang L. Ashley D. L. Watson C. H. (2011). Quantitative Analysis of Six Heterocyclic Aromatic Amines in Mainstream Cigarette Smoke Condensate Using Isotope Dilution Liquid Chromatography-Electrospray Ionization Tandem Mass Spectrometry. Nicotine Tob. Res. 13 (2), 120–126. 10.1093/ntr/ntq219 21173043

